# Ultrafine particles of *Ulmus davidiana* var. *japonica* induce apoptosis of gastric cancer cells via activation of caspase and endoplasmic reticulum stress

**DOI:** 10.1007/s12272-013-0312-2

**Published:** 2014-01-07

**Authors:** Joungjwa Ahn, Jong Suk Lee, Kyung Mi Yang

**Affiliations:** 1 Department of Food Science and Industry, Jungwon University, Goesan-gun, Chungbuk Korea; 2 Gyeonggi Biocenter, Gyeonggi Institute of Science and Technology Promotion, Suwon, Gyeonggi-do Korea; 3 Department of Biochemistry and Molecular Biology, Yonsei University College of Medicine, Yonsei-ro, Seodaemun-gu, Seoul, 120-752 Korea

**Keywords:** *Ulmus davidiana* var. *japonica*, Apoptosis, ER stress, Gastric cancer, Caspase

## Abstract

Small-sized particles are more suitable for targeted delivery and are therapeutically more effective than large-sized particles. In this study, we investigated the anticancer effects of ultrafine particles of *Ulmus davidiana* var. *japonica* (ufUJ) on human gastric cancer cell lines SNU-1, SNU-216, and SNU-484. ufUJ induced apoptosis by the proteolytic activation of caspase-9, caspase-6, and caspase-3 and cleavage of poly (ADP-ribose) polymerase. The expression levels of the endoplasmic reticulum stress-related protein BiP markedly increased after ufUJ treatment. BiP knockdown decreased ufUJ-induced cell death. ufUJ-induced apoptosis was inhibited by the caspase-3 inhibitor z-DEVD-fmk, caspase-6 inhibitor z-VEID-fmk, and caspase-9 inhibitor z-LEHD-fmk, and by siRNAs against caspases 3, 6, and 9. Gastric cancer cells did not show anchorage-independent growth in the presence of ufUJ. However, cells treated with caspase inhibitors showed an enhanced colony-forming ability. These findings may be helpful in the prevention of gastric cancer and in the development of functional foods.

## Introduction

Gastric cancer, which is the 14th most common cancer across the world, is one of the most aggressive types of malignant tumors and a major human health problem (Janunger et al. 2002). In many countries, most patients with gastric adenocarcinoma have advanced disease at diagnosis, and thus, the treatment options are limited. Many drugs are used for the clinical therapy for gastric cancer; however, specific systemic drugs need to be developed on the basis of an improved understanding of the gastric biology so as to improve the outcomes for patients with advanced disease. Therefore, antitumor drugs with a greater efficacy, lower toxicity, and no adverse effects are required (Singh et al. 2011; Cervantes et al. 2012). *Ulmus davidiana* var. *japonica* (UJ) has been planted widely in northern Japan and is used as a traditional medicine for its anti-inflammatory, anti-glycation, and anti-angiogenic activities; further, it exerts protective effects against glutamate-induced neurotoxicity and sepsis (Lee and Kim 2001; Lee et al. 2005; Choi et al. 2010; Jung et al. 2007; Zheng et al. 2011). Recently, a new technique has been developed for the production of ultrafine (smaller than 0.1 μm) particles of medicinal herbs. The particle size of medicinal materials is an important physical property that affects their pharmaceutical behavior (Yang et al. 2010). This ultrafine particle size is highly suitable for targeted delivery, and these particles are therapeutically more effective than large-sized particles (Lee et al. 2008; Choi et al. 2012) Because of their small size and large surface area, ultrafine particles have the capacity to carry and deposit high loads of active compounds deep into the target organs. Compared to large particles of therapeutic agents, ultrafine particles of these agents improve the therapeutic effects (Johnston et al. 2000; Lee et al. 2008). Ultrafine particles simultaneously induce apoptosis and proliferation in rat lung epithelial cells in a time- and dose-dependent manner (Sydlik et al. 2006). Lee et al. (2000) elucidated the effects of ultrafine particles produced by pulverization on in vitro tumor cell growth and in vivo proliferation of gastric epithelial cells.

Apoptosis is an essential physiological process that plays a key role in cancer prevention, treatment, and cell homeostasis. The caspase cascade system plays a vital role in the transduction of apoptotic signals. To date, three subfamilies of caspases have been identified; some of these caspases are involved in the activation of apoptosis while others mediate apoptosis induced by endoplasmic reticulum (ER) stress (Lawen 2003; Fan et al. 2005; Gorman et al. 2012). The stressed ER induces apoptosis via the unfolded protein response (UPR) pathway, which induces ER chaperones, and via the ER overload response pathway, which upregulates the expression of the glucose-regulated protein GRP78/BiP and phosphorylation of the eukaryotic initiation factor 2α (eIF2α) (Szegezdi et al. 2006).

In the present study, we investigated the molecular mechanisms underlying the antitumor effects of the ethanolic extract of pulverized particles of UJ (AM2) in gastric cancer cells by increasing the expression of ER markers and activation of caspases.

## Materials and methods

### Chemicals and reagents

The annexin V-fluorescein isothiocyanate (FITC) apoptosis detection kit (#556547) was purchased from BD Biosciences (Bedford, MA, USA). The primary antibodies for cleaved caspases 9, 6, and 3; poly (ADP-ribose) polymerase (PARP); tubulin; BiP; and secondary antibodies were purchased from Cell Signaling Technology (Beverly, MA, USA). Lactate dehydrogenase (LDH) cytotoxicity assay kits (#G1780) were purchased from Promega (Madison, WI, USA). The caspase inhibitor and caspase colorimetric assay kits were purchased from R&D Systems Inc. (Minneapolis, MN, USA). The HT TiterTACS assay kit (#4822-96-K) for quantitative detection of apoptosis was purchased from Trevigen (Gaithersburg, MD, USA). The water-soluble tetrazolium salt (WST-8) cell proliferation assay kit (#CK04-05) was obtained from Dojindo Laboratories (Kumamoto, Japan).

### Preparation of extracts

Dry powder of UJ was purchased from Kyungdong market in Seoul City, Korea. The powder of UJ was ground to obtain ultrafine particles by using an herbal medicine pulverizer (Delsa™ Nano; Beckman Coulter Inc., Brea, CA, USA). The ultrafine particles of UJ (ufUJ) were extracted twice with an equal volume of 80 % ethanol. The extracts were filtered through filter papers (3M, Paul, MN, USA) and evaporated using a Soxhlet apparatus. The ethanolic fractions were concentrated in a vacuum evaporator to obtain two fractions, namely AM1, extract of non-pulverized particles, and AM2.

### Cell lines and culture

We purchased three human gastric cancer cell lines SNU-1, SNU-216, and SNU-484 from the Korean Cell Line Bank (Seoul, Korea). All cells were tested for mycoplasma contamination and were maintained in Roswell Park Memorial Institute (RPMI) medium supplemented with 10 % fetal bovine serum (FBS). The cells were cultured in a 5 % CO_2_ incubator at 37 °C.

### Measurements of cell viability and LDH activity

Relative cell viability was measured using the WST-8 assay using the Cell Counting kit-8 (Dojindo). The activity of the soluble cytosolic enzyme LDH was determined by measuring the release of LDH into the culture medium by using a cytotoxicity assay kit according to the manufacturer’s protocol. The extracellular LDH activity induced by the AM1 and AM2 fractions was calculated as the relative concentration of LDH in the medium compared to that of the control.

### Propidium iodide/annexin V assay

Apoptosis was determined using the propidium iodide (PI)/annexin V detection method (BD Biosciences) by flow cytometry. The cells were treated with the AM1 and AM2 fractions and with ethanol as a vehicle control. After 24 h, the cells were harvested, and the assay was performed according to the manufacturer’s instructions.

### HT TiterTACS assay

The number of apoptotic cells was determined using an enzyme-linked immunosorbent assay (ELISA)-based HT TiterTACS assay kit according to the manufacturer’s instructions. The cells were fixed in a 3.7 % buffered formaldehyde solution for 5 min and postfixed in 100 % methanol for 20 min. To label the hydrated fixed cells, the cells were permeabilized with proteinase K solution. Endogenous peroxidase was quenched using hydrogen peroxide (H_2_O_2_), and the cells were washed with distilled water. Damaged DNA was tagged with a labeling mixture and streptavidin-horseradish peroxidase (HRP) solution, and then TACS-Sapphire was added to the wells to enable the detection of the apoptotic cells. The reaction was terminated using 5 % phosphoric acid and the absorbance was measured at 450 nm.

### Assessment of caspase activity

The three gastric cancer cell lines were seeded in six-well plates and were treated with the extracts at the specified concentrations. Specific caspase activity was determined using substrates of the color reporter molecule *p*-nitroaniline (*p*NA) specific for caspases. The lysates were centrifuged for 10 min at 12,000 rpm at 4 °C, and the supernatant was used for the assay. Caspase activity was measured using a microplate reader at 495 nm, and the activity was expressed as the fold increase compared to that of the control.

### Small interfering RNA duplex and transfection

Small interfering (si) RNA oligonucleotides (duplex sequence, 5′-GGAGCGCAUUGAUACUAGA-3′) and scrambled siRNA were used to target BiP. Caspase-3 siRNA, caspase-6 siRNA, and caspase-9 siRNA were purchased from Ambion (Austin, TX, USA). Cultured cells were transfected with the siRNAs using G-fectin (Genolution., Seoul, Korea) or Lipofectamine reagent (Invitrogen, Carlsbad, CA, USA) according to the manufacturer’s guidelines.

### Western blotting

The cells were exposed to AM1 or AM2 at concentrations ranging from 50 to 400 μg/ml, and the cells were harvested after 24 h. The cells were lysed with 1× RIPA buffer [20 mmol/l Tris–HCl (pH 7.4), 137 mmol/l NaCl, 10 % (v/v) glycerine, 1 % Triton X-100, and 2 mmol/l EDTA], and the protein concentration was determined using the Bradford assay (Bio-Rad Laboratories, Hercules, CA, USA). Samples were loaded onto a 12 % sodium dodecyl sulfate polyacrylamide gel electrophoresis (SDS-PAGE) gel. The proteins were transferred to a polyvinylidene fluoride (PVDF) membrane (Bio-Rad Laboratories) and blocked with 5 % (w/v) skim milk in Tris-buffered saline (TBS) for 2 h. Then, the samples were probed with antibodies against cleaved caspases 9, 6, and 3; cleaved PARP; BiP; and tubulin. The membranes were immunoblotted with the antibodies overnight and subsequently washed with 1× 0.05 % Tween 20 in phosphate-buffered saline (PBS) (TBST) for 40 min. The membranes were then incubated with mouse IgG HRP-conjugated secondary antibodies for 90 min, and the protein bands were detected using Pierce ECL-Plus chemiluminescence (Thermo Scientific, Schwerte, Germany).

### Anchorage-independent survival assay

To determine anchorage-independent growth, gastric cancer cells were treated with a caspase inhibitor for 2 h in the presence of 100 μg/ml of AM2, and the cells were detached using 0.05 % trypsin/EDTA (Sigma Chemical Co.). Detached cells (5 × 10^4^) were plated onto 60-mm plates containing a bottom layer of 0.6 % low-melting-temperature agar in RPMI and a top layer of 0.45 % agar in RPMI. The plates were incubated at 37 °C in a 5 % CO_2_ atmosphere, and the colonies were scored after 2 weeks of growth. Anchorage-independent cell survival was analyzed using crystal violet staining according to a method described previously.

## Results

### The effects of the ethanolic extracts of ufUJ on the morphology and plasma membrane integrity of gastric cancer cells

**Fig. 1:**
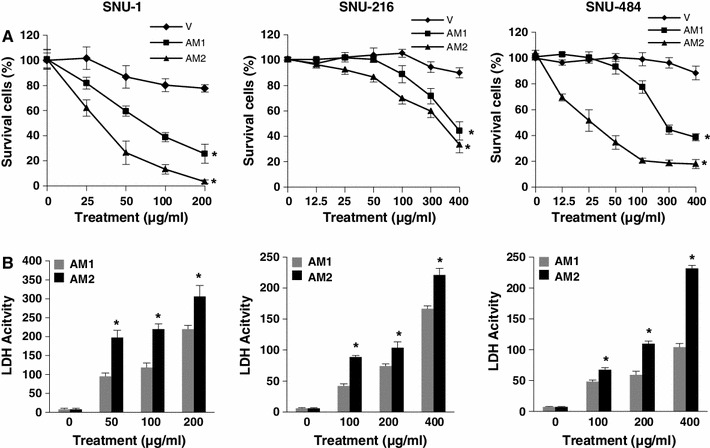
Influence of AM1 or AM2 on the viability of human gastric cancer cells. **a**, **b** Cells were treated with the specified concentrations of AM1, AM2, and vehicle for 24 or 48 h for the cell viability assay or for 36 h for the LDH assay. **a** Cell viability was determined using the WST-8 assay. *Filled diamond* vehicle, *filled square* AM1, *filled triangle* AM2. **b** LDH activity was determined by measuring the rate of reduction of a tetrazolium salt into highly colored formazan, the absorbance of which was measured at 490 nm by using a plate reader. The results are shown as the mean ± standard deviation (SD) of the data from three independent experiments (*P < 0.05 vs untreated control). *AM1* extracts from non-pulverized particles, *AM2* extracts from pulverized particles, *LDH* lactate dehydrogenase

We examined the effects of AM1 and AM2 (200 μg/ml for 24 h) on the morphological characteristics of SNU-216 and SNU-484 cells using an inverted microscope. The AM2 extract induced membrane blebbing, cell shrinkage, and formation of floating cells (data not shown). These morphological changes indicated that treatment with ufUJ induced apoptosis. Further, we investigated the effect of AM1 and AM2 on the growth of the three gastric cancer cell lines; we used the trypan blue dye exclusion test to directly monitor the survival rate. Cell viability decreased in the presence of both extracts. Treatment with different concentrations (0, 25, 50, 100, and 200 μg/ml of AM1 or 0, 12.5, 25, 50, 100, 300, and 400 μg/ml of AM2 for 24 h) of AM1 and AM2 decreased the survival of SNU-1 (AM1, 25.6 ± 7.5 %; AM2, 3.3 ± 1.4 %), SNU-216 (AM1, 44.3 ± 7.1 %; AM2, 33.3 ± 6.3 %), and SNU-484 (AM1, 38.6 ± 2.7 %; AM2, 17.9 ± 3.4 %) cell lines compared to that of the vehicle-treated control (Fig. 1a). The half-maximal inhibitory concentration (IC_50_) of AM1 and AM2 in the SNU-1 cells were ~75 and 37 μg/ml, respectively. The IC_50_ values of AM1 and AM2 in the SNU-216 cells were ~370 and 320 μg/ml, respectively, and the IC_50_ values of AM1 and AM2 in SNU-484 cells were ~280 and 25 μg/ml, respectively.

We examined LDH activity to determine whether AM1 or AM2 induced a loss of membrane integrity. Determination of the plasma membrane integrity is one of the most straightforward ways to measure cell viability and cytotoxic consequences. The activity of the soluble cytosolic enzyme LDH is commonly measured to determine plasma membrane integrity. If cell membranes are damaged, LDH is released into the culture medium because of the loss of membrane integrity. Compared to treatment with AM1 or control, treatment with AM2 showed a significant increase in LDH release into the surrounding culture medium (Fig. 1b). After 24 h of exposure to AM1 and AM2, a dose-dependent increase was observed in the amount of LDH released into the culture medium. Unlike, vehicle-treated controls, LDH activity was detected in SNU-1 (AM1, 219.6 ± 29.1 %; AM2, 306.0 ± 10.0 %), SNU-216 (AM1, 166.3 ± 4.7 %; AM2, 221.0 ± 10.8 %), and SNU-484 (AM1, 104.0 ± 6.0 %; AM2, 231.3 ± 5.1 %) cells treated with AM1 and AM2. The maximum LDH activity in the cells exposed to 200 or 400 μg/ml of AM2 was higher than that of the cells exposed to AM1. These results suggest that compared to treatment with AM1, treatment with AM2 caused an increase in the number of damaged cells because of an increased efficiency in the delivery of ufUJ.

### Apoptosis induced by ufUJ

**Fig. 2:**
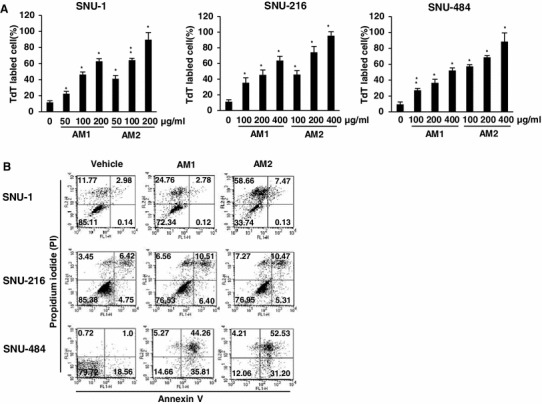
Induction of apoptosis as well as necrosis in human gastric cancer cells by treatment with the AM1 and AM2. **a** The human SNU-1, SNU-216, and SNU-484 gastric cancer cell lines were treated with the indicated concentrations of AM1 and AM2 for 24 h. Damaged DNA was tagged with TdT labeling. **b** The three cell lines were incubated for 24 h with 200 μg/ml of AM1 and AM2. Apoptotic cells were evaluated by their profile of annexin-V staining in the X-axis and PI in the Y-axis. The results are shown as the mean ± SD of the data from three independent experiments (*P < 0.05; **P < 0.01 vs untreated control). *AM1* extracts from non-pulverized particles, *AM2* extracts from pulverized particles

Previous studies showed that AM2 decreased the plasma membrane integrity of the three gastric cancer cell lines. To investigate the mechanisms underlying the effects of AM1 and AM2 in these cancer cell lines, we identified the apoptotic gastric cancer cells by TdT labeling (Fig. 2a). The vehicle ethanol did not affect DNA damage; however, apoptosis was higher in AM2-treated gastric cells than in AM1-treated cells after 24 h (Fig. 2a). Treatment of SNU-1 cells with AM1 at concentrations of 50, 100, and 200 μg/ml induced 22.4 ± 2.8, 46.3 ± 3.2, and 62.8 ± 3.2 % DNA fragmentation, respectively, and treatment with AM2 at these concentrations induced 41.1 ± 3.8, 64.2 ± 2.4, and 89.7 ± 8.9 % DNA fragmentation, respectively. Treatment of SNU-216 cells with 100, 200, and 400 μg/ml of AM1 induced 35.4 ± 6.2, 45.4 ± 6.2, and 63.7 ± 5.4 % DNA fragmentation, respectively, and treatment with AM2 at these concentrations induced 45.8 ± 5.0, 74.1 ± 7.6, and 95.4 ± 5.0 % DNA fragmentation, respectively. On the basis of these patterns of DNA damage shown by the TdT labeling assay, cells with AM2-induced apoptosis were further characterized by PI/annexin V staining. The 3 cell lines were treated with AM1 and AM2 at 200 μg/ml and ethanol as a control for 24 h. Our data showed that treatment with the vehicle had little effect on apoptosis, while treatment with 200 μg/ml of AM1 or AM2 induced a slight apoptotic effect in the SNU-216 cells. The number of apoptotic cells in the SNU-1 cell line treated with AM2 (66.2 %) was markedly higher than that in the SNU-1 cell line treated with AM1 (27.66 %, Fig. 2b). Under the same culture conditions as those used for SNU-1 cells, SNU-484 cells treated with AM1 showed 85.34 % apoptosis, and those treated with AM2 showed 87.9 % apoptosis. These results indicate that AM2 showed a stronger apoptotic effect than AM1.

### ufUJ induces the cleavage of PARP, caspase activation, upregulation of BiP, and phosphorylation of eIF2α

**Fig. 3:**
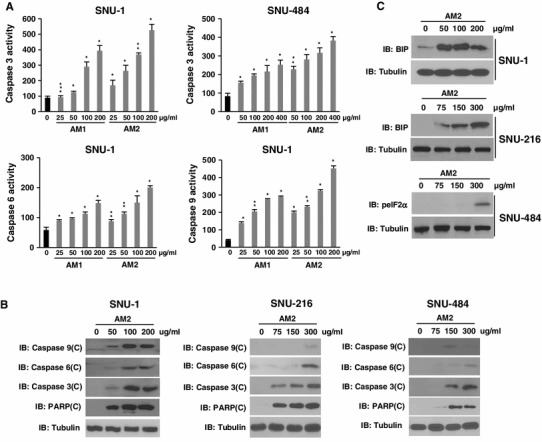
Caspase activity and ER stress biomarkers induced by AM1 or AM2 treatment. **a** Caspase activity was analyzed after treating cells with the indicated concentrations of the extracts for 24 h. Quantitative measurement of caspase-9, caspase-6, and caspase-3 activity was performed via the detection of *p*NA released from their respective colorimetric substrates. **b**, **c** Human SNU-1, SNU-216, and SNU-484 gastric cancer cells were cultured for 24 h with the indicated concentrations of AM2 and immunoblotted with cleaved caspase-9, cleaved caspase-6, cleaved caspase-3, cleaved PARP, BiP, peIF2α, and tubulin antibodies. The results are shown as the mean ± SD of the data from three independent experiments (*P < 0.05; **P < 0.01; ***P < 0.001 vs untreated control). *AM1* extracts from non-pulverized particles, *AM2* extracts from pulverized particles

To investigate the involvement of caspases in AM2-mediated apoptosis, we treated the cells with AM1 and AM2 (0, 25, 50, 100, and 200 μg/ml or 0, 50, 100, 200, and 400 μg/ml for 24 h), prepared cell extracts, and examined the protease activity using a colorimetric substrate specific for caspase-9, caspase-6, and caspase-3 (Fig. 3a). The degradation products of BiP, cleaved caspases 9, 6, and 3, and PARP were detected by immunoblotting (Fig. 3b, c). Activation of caspases is a crucial mechanism for the induction of apoptosis, and caspase-9, caspase-6, and caspase-3 are key proteases that execute or activate apoptosis (Fan et al. 2005). UJ activated caspase-3 in a dose dependent manner in SNU-1 and SNU-484 cells (Fig. 3a, b). In addition, our data showed that the increase in caspase-3 activation induced by AM2 was greater than that induced by AM1 in both cells. Similarly, compared to treatment with AM1, treatment with AM2 induced a dose-dependent and greater increase in the activation of caspase-6 and caspase-9. Further, we performed immunoblot analysis of the apoptosis-inducing molecules in the cell lysate obtained from AM2-treated SNU-1, SNU-216, and SNU-484 cells. We used antibodies that specifically recognize the cleaved forms of the caspases and PARP, and the biomarkers of ER stress, BiP, and eIF2α. Marked increases were observed in the expression levels of cleaved caspases 3, 6, 9, and cleaved PARP in the SNU-1 and SNU-484 cells after treatment with AM2, whereas we were unable to detect any cleaved caspase-9 in the SNU-216 cells. In addition, the levels of ER stress markers BiP and phosphorylated eIF2α (peIF2α), which are important markers associated with ER stress-induced apoptosis, increased after treatment with AM2. These results indicated that AM2-induced apoptosis of gastric cancer cells was associated with the proteolytic activation of caspase-9, caspase-6, and caspase-3; PARP cleavage; and the expression of ER stress modulators such as BiP and peIF2α.

### Inhibition of caspases and BiP suppresses ufUJ-induced apoptosis

**Fig. 4:**
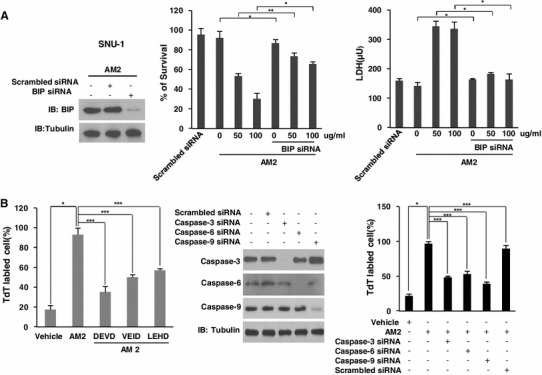
Knockdown of BiP and caspase-9, caspase-6, and caspase-3 inhibits apoptosis induced by AM2 in human gastric cancer SNU-1 cells. **a** Cells were plated in 6-well plates and allowed to reach 50 % confluence on the next day. A siRNA against BiP was transfected into SNU-1 cells using lipofectamine. A scrambled siRNA was used as a control. After 30 h of transfection, the cells were exposed to the indicated concentrations of AM2 for 24 h. **b** Cells were seeded in 60-mm plates and allowed to reach 50 % confluence on the next day. The siRNAs against caspase-3, caspase-6, and caspase-9 were transfected into SNU-1 cells using G-fectin. A scrambled siRNA was used as a control. After 30 h of transfection, the cells were exposed to 50 μg/ml of AM2 for 24 h. Apoptotic cells were quantified with a TdT tagging assay. The results are shown as the mean ± SD of the data from three independent experiments (*P < 0.05; **P < 0.01; **P < 0.001 vs untreated control or AM2 alone). *AM1* extracts from non-pulverized particles, *AM2* extracts from pulverized particles

To further confirm that the induction of BiP and the activation of caspases were implicated in ufUJ-induced apoptosis, and that treatment of SNU-1 cells with AM2 resulted in apoptosis via the activation of these 3 caspases, we used siRNA duplexes to knockdown the expression of BiP and the 3 caspases in SNU-1 cells. Because the caspase-3 inhibitor z-DEVD-fmk, caspase-6 inhibitor z-VEID-fmk, and caspase-9 inhibitor z-LEHD-fmk inhibit caspase activation, we evaluated whether these caspase inhibitors could block AM2-mediated apoptosis. Cell survival was measured using the WST-8 assay, and plasma membrane integrity in the gastric cells was measured using the LDH assay. Then, we performed HT TiterTACS assay to examine the characteristics of the apoptotic event. We measured the efficacy of the siRNA silencing of caspase-3, caspase-6, caspase-9, and *BiP* gene expression using western blotting. These results were further confirmed with an anchorage-independent cell growth assay, in which the SNU-484 cells were incubated with a caspase inhibitor and AM2. Compared to cells transfected with scrambled control siRNAs, those transiently transfected with BiP and caspase-suppressing siRNAs substantially decreased the expression levels of BiP and the 3 caspases (Fig. 4a, b). The WST-8 reduction assay measures the cellular reductive capacity through the extracellular reduction of WST-8 tetrazolium salt by an electron-coupling reagent in cultures on the basis of quantification of the ATP present, which results in the formation of a highly light-absorbent formazan product. Our results show that compared to the AM2 treated control cells and scrambled siRNA transfected control cells, cells treated with AM2 and transfected with the siRNA duplex showed an increased number of metabolically active cells (Fig. 4a). The amount of LDH released after AM2 treatment under transfection with the siRNA duplexes in the SNU-1 cells was significantly lower than that after AM2 treatment (Fig. 4a). These findings were consistent with the significant decrease in the AM2-induced apoptosis observed after co-cultivation with inhibitor of caspase-9, caspase-6, and caspase-3, which decreased the number of TdT-labeled cells (Fig. 4b). The siRNAs against the 3 caspases suppressed AM2-induced apoptosis, which were consistent with the above-mentioned findings (Fig. 4b). These results suggested that AM2 induced apoptosis by increasing the levels of active caspase-9, caspase-6, caspase-3, and BiP in the gastric cancer cells.

### Inhibition of caspase increases anchorage-independent cell growth in response to ufUJ treatment

**Fig. 5:**
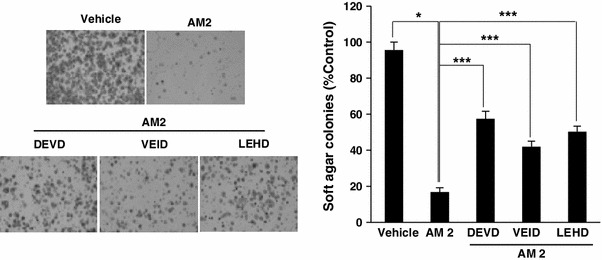
Increased anchorage-independent growth of AM2-treated gastric cancer cells by DEVD, VEID, or LEHD. **a** Cells were treated with the vehicle and 50 μg/ml of AM2 in the presence or absence of caspase inhibitors (DEVD, 30 μM; VEID, 30 μM; LEHD, 30 μM). After 2 weeks of culture, the cells were stained with crystal violet dye. The images of the stained colonies were obtained using a microscope under ×100 magnification. **b** The number of colonies expressed as a percentage of the vehicle-treated control. The results are shown as the mean ± SD of the data from three independent experiments (*P < 0.05; **P < 0.001 vs untreated control or AM2 alone). *AM1* extracts from non-pulverized particles, *AM2* extracts from pulverized particles

As was shown above, AM2 induces caspase activation and biochemical markers of apoptosis in gastric cancer cells. We measured anchorage-independent cell growth after AM2 treatment. Anchorage-independent cell growth has been associated with the metastatic potential of cancer cells (Mori et al. 2009). In addition, we investigated whether caspase-9, caspase-6, and caspase-3 inhibitors could restore the growth of the colonies. Treatment with AM2 decreased the anchorage-independent growth of the SNU-484 cells indicated by their ability to grow as colonies in soft agar. The vehicle-treated SNU-484 cells formed numerous colonies, and the number of colonies markedly decreased after AM2 treatment (Fig. 5). However, the addition of caspase-9, caspase-6, and caspase-3 inhibitors increased the colony formation in soft agar containing AM2. AM2 treatment induced a decrease in the number of SNU-484 colonies compared to those observed after treatment with vehicle control, and the addition of 30 μg/ml each of DEVD, VEID, and LEHD to the AM2-treated SNU-484 cells significantly increased the numbers of colonies. Results similar to those obtained with SNU-484 cells were obtained with the SNU-1 cells (data not shown). These results indicate that AM2 inhibits the anchorage-independent growth of human gastric cancer cells. In addition, AM2 induced apoptosis and stimulated caspase-9, caspase-6, and caspase-3 activities under anchorage-independent conditions consistent with the above-mentioned findings.

## Discussion

Several studies have shown that the compounds isolated from UJ exert pharmacological activities in various types of cancer cells and in animal models. Therefore, UJ appears to have great potential as a medicinal plant, and compounds isolated from this plant have chemopreventive properties. Our previous studies showed that treatment with ufUJ induced morphological changes in cancer cells in a dose-dependent manner, which leading to cell shrinkage and DNA ladder formation associated with apoptosis (data not shown). However, the in vitro apoptotic signaling mechanisms underlying the anticancer effect of UJ have not been completely elucidated thus far. In this study, we examined the anticancer effect of an ethanolic extract of ufUJ particles in gastric cancer cells and the mechanisms underlying its apoptotic effect. Our results were consistent with the findings of metabolically active cell detection assay, plasma membrane integrity assay, TdT labeled cell detection, apoptotic cell sorting by fluorescence-activated cell sorting (FACS), caspase activity assay, immunoblotting, and soft agar assay, all of which showed that ufUJ induced ER stress/caspase activation-mediated apoptosis or necrotic cell death of gastric cancer cells.

The results of our study showed that the ethanolic extract of ufUJ caused a loss of plasma membrane integrity and induced ER stress and apoptosis in human gastric cancer cells through the upregulation of BiP and activation of caspase-9, caspase-6, and caspase-3, which play an important role in the apoptotic process (Fig. 4, 5). Previous studies indicate that when UJ is pulverized into ultrafine particles, most of the particles can pass through the cell membrane by diffusion and penetration processes (Xu et al. 2001; Yang and Gai 2007; Ma et al. 2009). A pulverization technique has been developed to produce ultrafine particles of medicinal herbs, which range from 0.1 to 1 μm. This ultrafine particle size is ideal for targeted delivery and causes apoptosis in human cancer cells (Lee et al. 2008; Choi et al. 2012). Our results suggest that the ufUJ particles easily penetrate the cell membrane and mediate apoptosis by inducing cellular ER stress in human gastric cancer cells.

Apoptosis is an essential physiological process that plays a critical role in development and cell homeostasis. Apoptosis represents a distinct set of biochemical and physical changes involving the nucleus, cytoplasm, and plasma membrane (Lawen 2003). Because cytoskeletal changes and LDH release are directly associated with apoptosis and the concomitant DNA damage, we measured the number of metabolically active cells and TdT-labeled DNA after ufUJ treatment. Many chemical reagents, oxidative stress, alcohol, and other cellular stresses can trigger ER stress-induced apoptosis (Gorman et al. 2012). Caspases, which are closely associated with apoptosis, are aspartate-specific cysteine proteases regulated by the Bcl-2 family, calpain, and Ca^2+^ ions (Fiandalo and Kyprianou 2012; Smith and Schnellmann 2012). The intraluminal ER chaperone BiP is a member of the heat shock protein family and is a potent regulator of the ER stress mediators ATF6, PERK, and IRE1. The eIF2 is regulated by a mechanism involving both guanine nucleotide exchange and phosphorylation at the α-subunit, which is a target for a number of serine kinases that phosphorylate serine 51 (Lee et al. 2008; Teske et al. 2011).Our data showed that AM2 treatment was able to upregulate BiP expression in dose-dependent manner in gastric cancer cells. Usually, severe ER stress provokes a complex network of interacting leading to apoptosis. Also, AM2-induced apoptosis involves the activation of caspase-9, caspase-6, and caspase-3. The activities of caspases were enhanced significantly in a dose-dependent manner during AM2 treatment (Fig. 3). The roles of BiP and caspase in AM2-induced apoptosis were analyzed using a synthetic chemically modified siRNA delivery system as well as the selective caspase inhibitors DEVD, VEID, and LEHD. BiP knockdown and caspases inhibition experiment in the AM2 treated gastric cells resulted in an increased cell survival, the release of LDH into the medium, and DNA damage indicative of apoptosis. These results are consistent with results observed with inhibitors of caspase-9, caspase-6, and caspase-3 in SNU-1 cells, which suggested that BiP and the 3 caspases examined in this study may be distinct targets in the apoptotic effect of AM2 in gastric cancer cells. In addition, we showed an effect of AM2 on colony formation in soft agar. Anchorage-independent cell growth inhibition has been associated with anti-metastatic activity in cancer cells (Mori et al. 2009). Gastric cancer cells did not show anchorage-independent growth in the presence of AM2. Thus, AM2 strongly inhibited caspase-induced anchorage-independent growth of gastric cancer cells. Tandem mass analysis in the negative ion mode indicated that the ultra-fine UJ particles contained a flavonoid compound, catechin-7-*O*-xyloside (data not shown). In a recent study, some active flavonoids were quantified using high-performance liquid chromatography (HPLC) using various solvent extracts obtained from UJ (Lee et al. 2008). This finding indicates that some constituents in the extract are critical for antioxidant actions. Therefore, AM2-containing flavonoids, specifically catechin-7-*O*-xyloside, have the potential as effective and safe agents for the prevention of cancer.

In summary, we have identified novel mechanisms underlying the apoptotic effect of ultrafine particles of UJ. The effect of certain constituents of ufUJ particles easily penetrate the cell membrane and mediate apoptosis in gastric cancer cells through the regulation of ER stress markers such as BiP expression and eIF2α phosphorylation and apoptosis-related caspases. Further studies should be performed to isolate a specific active compound from UJ that is effective for the treatment of cancer.

## Abbreviations

UJ: *Ulmus davidiana* var. *japonica*
ufUJ: Ultrafine particles of *Ulmus davidiana* var. *japonica*
ER: Endoplasmic reticulum
WST-8: Water-soluble tetrazolium salt
LDH: Lactate dehydrogenase
PI: Propidium iodide
PARP: Poly(ADP-ribose) polymerase
eIF2α: Eukaryotic initiation 37 factor 2α
LC–MS: Liquid chromatography–mass spectrometry
UHPLC: Ultra-high performance liquid chromatography
SEM: Scanning electron microscopy
